# Visual Epilepsy Mimicking Migraine Aura Leading to Delayed Diagnosis of an Occipital Cavernoma: A Case Report

**DOI:** 10.7759/cureus.106768

**Published:** 2026-04-10

**Authors:** Manal Touilite, Yasmina Zakaria, Mohamed Chraa, Najib Kissani, Nissrine Louhab

**Affiliations:** 1 Department of Neurology, Mohamed VI Hospital University, Cadi Ayyad University, Marrakech, MAR

**Keywords:** cerebral cavernoma, cerebral cavernous malformation, diagnostic delay, electroencephalography (eeg), migraine with aura, occipital lobe seizure, visual epilepsy

## Abstract

Cerebral cavernous malformations, also known as cavernomas, are uncommon vascular malformations that may present with seizures, headache, or focal neurologic deficits depending on lesion location. Occipital cavernomas are rare and may manifest with positive visual phenomena, thereby mimicking migraine aura and delaying diagnosis. We report the case of a 54-year-old woman who presented in March 2018 with recurrent brief visual episodes described as flashing lights, colored shapes, scotomas, and distortion of object contours, associated with a persistent headache. She was initially treated for migraine with aura for approximately two years without clinical improvement. Repeated electroencephalography (EEG) later demonstrated rare focal epileptiform discharges in the left temporo-occipital region. Brain magnetic resonance imaging (MRI) revealed a rounded left occipital cortico-subcortical lesion measuring 17 x 16.5 x 16 mm, with imaging features suggestive of a cavernoma and without significant mass effect or perilesional edema. Carbamazepine was gradually titrated to 800 mg/day orally in two divided doses, with complete resolution of visual seizures. No surgical treatment was required, and the patient remained seizure-free during long-term follow-up. This case highlights the diagnostic overlap between visual epilepsy and migraine aura in patients with occipital cavernoma. It also underscores the importance of early MRI and EEG in patients with atypical or treatment-resistant visual symptoms, as timely recognition may permit effective medical management and avoid prolonged diagnostic delay.

## Introduction

Cerebral cavernous malformations (CCMs), also referred to as cavernous angiomas, are occult vascular malformations characterized by enlarged, thin-walled sinusoidal capillaries without intervening brain parenchyma [1]. They represent 5% to 13% of all vascular malformations of the central nervous system and may occur sporadically or in familial forms [1]. They are relatively common neurovascular lesions, with an estimated prevalence ranging from 0.4% to 0.9% in the general population [2].

Clinically, CCMs exhibit a broad spectrum of presentations, ranging from asymptomatic incidental findings (48%) to symptomatic cases, most commonly manifesting as seizures, which account for more than 25% of cases, intracranial hemorrhage, or focal neurological deficits, depending on lesion location [2]. Approximately 70-80% of CCMs are found in the supratentorial compartment, where seizures are the most frequent clinical manifestation. Occipital lobe involvement is rare, representing only 4.3% of supratentorial cases [2].

When located in the occipital region, CCMs can present with brief positive or negative visual symptoms, including flashes, colored phenomena, scotomas, or visual distortion. Such symptoms may closely mimic migraine with aura, especially when headache coexists, representing an important differential diagnosis and a potential source of diagnostic delay [3]. Other relevant differential diagnoses include occipital lobe epilepsy, posterior circulation ischemia, and structural brain lesions such as tumors or vascular malformations.

From a diagnostic perspective, CCMs are angiographically occult lesions, making magnetic resonance imaging (MRI) the gold standard for detection; they typically demonstrate a characteristic “popcorn-like” appearance with a hemosiderin rim. Electroencephalography (EEG) plays a complementary role in identifying associated epileptic activity, particularly in patients presenting with seizure-like symptoms [2].

Management strategies for CCMs remain a subject of debate. They may include conservative treatment with antiepileptic drugs or surgical resection, depending on symptom severity, lesion location, and the risk of complications such as hemorrhage or drug-resistant epilepsy [3].

This case is reported to highlight the diagnostic challenges posed by occipital cavernomas presenting with visual symptoms mimicking migraine aura and to emphasize the importance of early neuroimaging and EEG in atypical or treatment-resistant cases, ultimately supporting improved patient outcomes.

## Case presentation

A 54-year-old woman presented in March 2018 with persistent headaches and intermittent visual disturbances. She described brief attacks of photopsias, chromatopsias, scotomas, and metamorphopsias. These episodes lasted from a few seconds to a few minutes, occurred intermittently, and had no clear trigger. The frequency of visual episodes was variable, occurring almost daily during some periods, while at other times they were more sporadic and infrequent. The visual symptoms occurred either after the onset of headache or independently of it and lasted no longer than one hour. Her headache was dull and persistent, sometimes associated with mild photophobia, but without nausea or vomiting.

Her medical history was otherwise unremarkable; however, she reported a maternal history of migraine. On examination, she was hemodynamically stable and in no acute distress. Neurologic examination showed no focal deficit. Cranial nerves were intact, motor and sensory examination was normal, and brisk deep tendon reflexes were noted. Cerebellar examination was normal, and no meningeal signs were observed. Funduscopic examination showed no papilledema, retinal hemorrhage, or exudate. The remainder of the physical examination was unremarkable.

**Figure 1:**
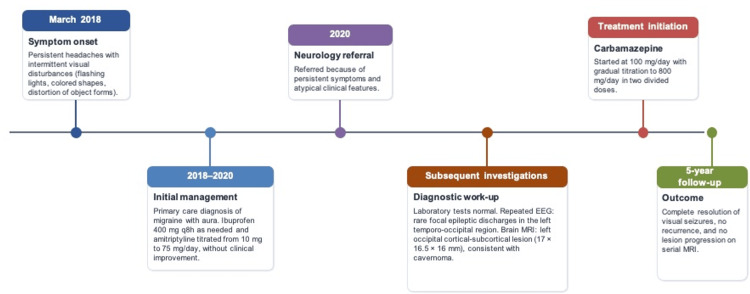
Timeline of clinical course and management Chronological summary of symptom onset, diagnostic workup, treatment initiation, and long-term outcome in a patient with visual seizures secondary to an occipital cavernoma. MRI: magnetic resonance imaging, EEG: electroencephalography

At initial evaluation, the clinical presentation was interpreted as migraine with aura. She received symptomatic treatment with oral ibuprofen 400 mg every eight hours as needed and preventive treatment with oral amitriptyline, initiated at 10 mg nightly and progressively titrated to 75 mg/day. Despite treatment escalation and adequate adherence, no meaningful clinical improvement was observed over approximately two years. Due to the prolonged diagnostic course and multiple therapeutic steps, a timeline summarizing the clinical evolution, investigations, and management is presented (Figure 1).

Because of persistent symptoms despite optimized migraine therapy, she was referred for neurologic reassessment. Given the atypical nature of the visual episodes and lack of response to migraine therapy, other differential diagnoses were considered, including occipital lobe epilepsy, posterior circulation ischemia, and structural brain lesions, prompting further neuroimaging and EEG studies.

Laboratory investigations, including complete blood count, serum electrolytes, renal and liver function tests, and thyroid function tests, were within normal limits. Inflammatory markers, including ESR and CRP, were likewise unremarkable (Table 1).

**Table 1:** Summary of laboratory investigations AST: aspartate aminotransferase, ALT: alanine aminotransferase, TSH: thyroid-stimulating hormone, CRP: C-reactive protein, ESR: erythrocyte sedimentation rate

Test	Result	Unit	Reference range
Hemoglobin	13.4	g/dL	12.0–16.0
White blood cell count	6.9	×10⁹/L	4.0–10.0
Platelet count	265	×10⁹/L	150–400
Sodium	138	mmol/L	135–145
Potassium	4.2	mmol/L	3.5–5.1
Creatinine	74	µmol/L	45–90
Urea	4.4	mmol/L	2.5–7.5
AST	24	U/L	10–35
ALT	19	U/L	10–35
TSH	2.3	mIU/L	0.4–4.0
CRP	1	mg/L	<5
ESR	5	mm/hr	0–20

**Figure 2:**
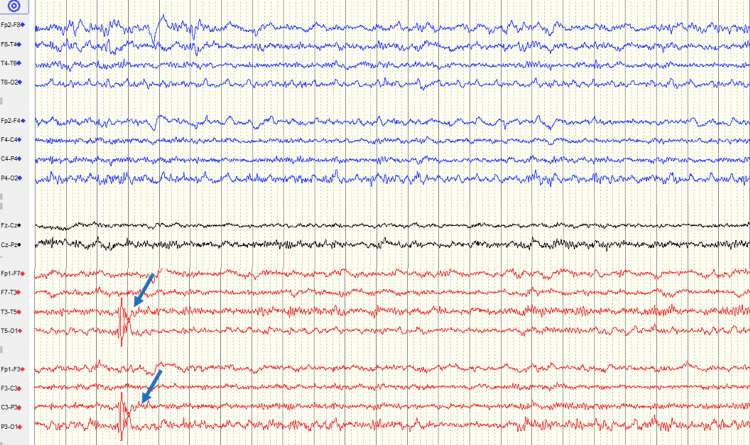
Interictal EEG Interictal EEG showing rare focal epileptiform discharges over the left temporo-occipital region, involving the T3–T5, T5–O1, and P3–O1 derivations (blue arrows), supporting the diagnosis of focal visual epilepsy. The EEG was recorded using a standard 10–20 system with a longitudinal bipolar montage, sensitivity of 7 µV/mm, time base of 30 mm/s, LFF of 1 Hz, HFF of 70 Hz, and a 50 Hz notch filter. The discharges were interictal and did not correlate with clinical events during the recording. EEG: electroencephalography, LFF: low-frequency filter, HFF: high-frequency filter

Repeated EEG recordings demonstrated rare focal epileptiform discharges over the left temporo-occipital region, supporting the diagnosis of focal visual seizures. The EEG was recorded using a standard 10-20 system with a longitudinal bipolar montage, sensitivity of 7 µV/mm, time base of 30 mm/s, low-frequency filter (LFF) of 1 Hz, high-frequency filter (HFF) of 70 Hz, and a notch filter at 50 Hz. The epileptiform discharges were interictal and showed no direct clinical correlation during recording (Figure 2).

**Figure 3:**
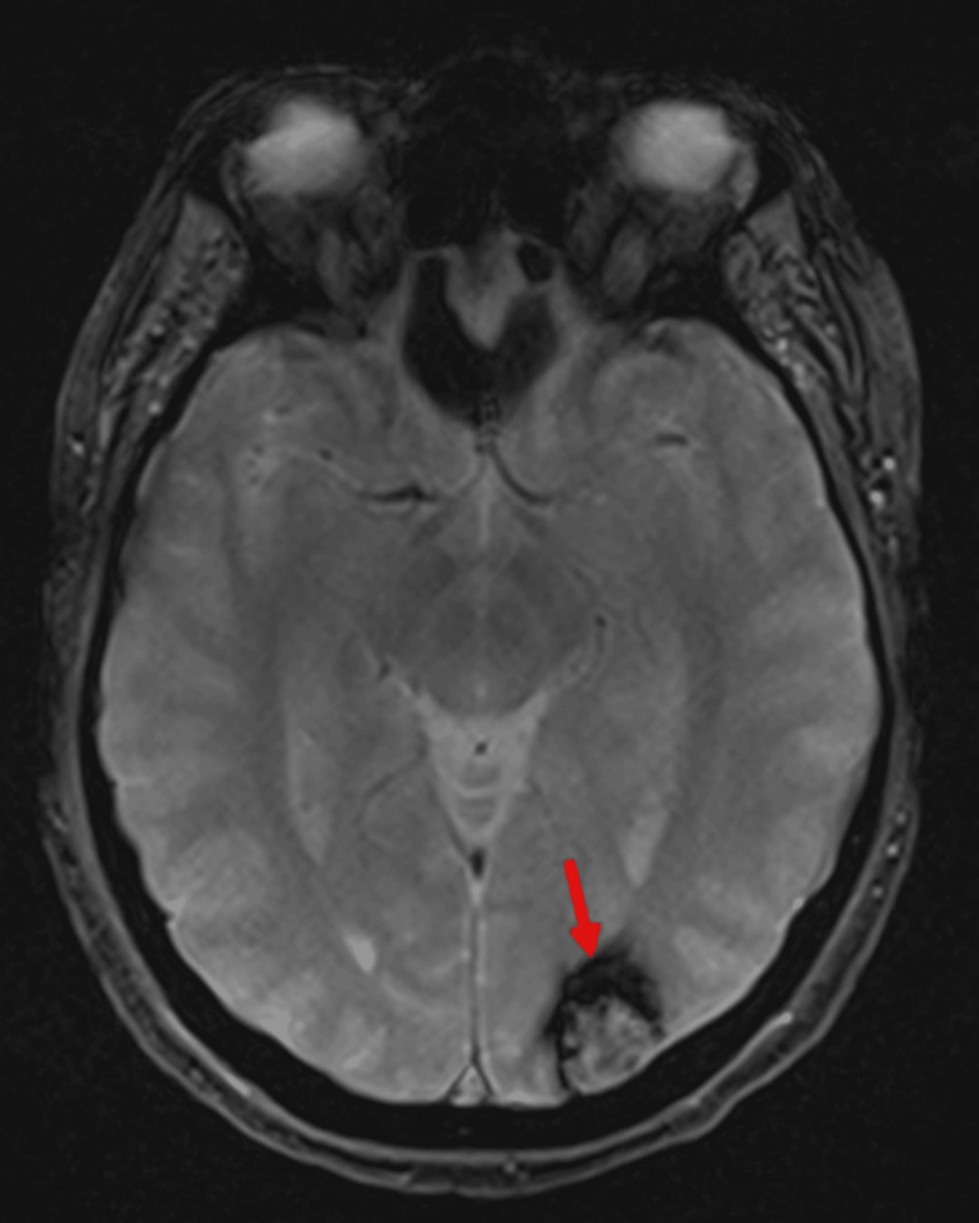
MRI of the brain Axial T2*/GRE MRI demonstrating a rounded left occipital cortico-subcortical lesion measuring 17 × 16.5 × 16 mm, with heterogeneous signal intensity and a peripheral hypointense hemosiderin rim producing a blooming artifact, consistent with a Zabramski type II cavernous malformation (red arrow). No additional structural abnormalities are identified. MRI: magnetic resonance imaging, GRE: gradient-recalled echo

Brain MRI revealed a rounded left occipital cortico-subcortical lesion measuring 17 × 16.5 × 16 mm, demonstrating heterogeneous signal intensity with a peripheral hemosiderin rim on susceptibility-sensitive sequences. On T2-weighted gradient-recalled echo (GRE) imaging, the lesion exhibited a characteristic blooming artifact, consistent with a Zabramski type II cavernous malformation. No additional structural brain abnormalities were identified (Figure 3).

Antiseizure treatment was then initiated with oral carbamazepine at 100 mg/day, followed by gradual weekly titration to 800 mg/day in two divided doses. Clinical tolerance and routine laboratory parameters were monitored during dose escalation, and treatment was well tolerated. After reaching the target dose, the visual episodes resolved completely.

Surgical intervention was not pursued because the patient achieved full symptomatic control with medical therapy, had no neurologic deficit, and showed no radiologic evidence of lesion progression. During follow-up extending beyond five years, she remained seizure-free, reported the complete disappearance of visual symptoms, and experienced marked improvement in daily functioning and quality of life. Serial MRI examinations showed lesion stability, and no treatment resistance or neurologic deterioration was documented.

## Discussion

CCMs, also referred to as cavernous angiomas, are occult vascular malformations characterized by enlarged, thin-walled sinusoidal capillaries without intervening brain parenchyma [4]. They are relatively common neurovascular lesions, with an estimated prevalence ranging from 0.16% to 0.5% in the general population. While 20% to 50% of cases remain asymptomatic and are often detected incidentally on MRI, symptomatic patients typically present with seizures (50%), hemorrhage (25%), or focal neurological deficits (25%) [5].

Although CCMs can occur anywhere within the central nervous system, approximately 75% to 80% are located in the supratentorial compartment, most commonly affecting the subcortical regions of the frontal and temporal lobes [4,6]. Occipital locations, as observed in this patient, are less frequent but are more likely to present with visual phenomena due to their proximity to the visual cortex [2].

A notable aspect of this case is the two-year diagnostic delay, during which the patient was managed for migraine with aura. CCMs are frequently underdiagnosed, as they are angiographically occult and may not be detected on routine computed tomography scans [4]. In this patient, intermittent visual disturbances, characterized by flashing lights and distortion of object shapes, were initially interpreted as migrainous aura. Similar diagnostic challenges have been reported in the literature, where CCM-related symptoms may mimic primary headache disorders, particularly migraine [4]. This overlap contributes to potential misdiagnosis and delayed recognition. In this context, MRI, particularly high-resolution 3T MRI, remains the gold standard for detection, typically demonstrating the characteristic “popcorn-like” lesion with a peripheral hypointense hemosiderin rim [7,8].

Seizures represent the most common clinical manifestation of supratentorial CCMs, occurring in up to 70% of symptomatic patients [9]. The epileptogenicity of these lesions is not intrinsic, as CCMs lack neuronal elements; rather, it is attributed to secondary changes in the surrounding brain tissue. Recurrent microhemorrhages lead to hemosiderin and iron deposition, which induce oxidative stress, reactive gliosis, and inflammatory changes, ultimately resulting in cortical hyperexcitability [9]. In the present case, the rare focal epileptiform discharges identified in the left temporo-occipital region are consistent with the lesion’s location and correlate well with the patient’s visual symptoms, supporting the diagnosis of visual epilepsy secondary to an occipital cavernoma.

The management of CCMs remains a subject of ongoing debate, particularly regarding the optimal timing and indications for intervention [6]. A significant point of contrast between our findings and existing literature lies in the therapeutic approach. Numerous retrospective studies and reviews of supratentorial cavernomas advocate microsurgical resection as the gold standard for symptomatic lesions, especially in patients with epilepsy, due to its potential to achieve definitive seizure control and reduce the risk of hemorrhage [10]. Furthermore, up to 40% of patients may progress to drug-resistant epilepsy if left untreated [10]. In contrast, our patient achieved sustained seizure freedom for more than five years with exclusive medical therapy using carbamazepine. While some studies support an initial conservative approach with antiepileptic drugs, particularly in patients with a single seizure or lesions located in eloquent brain areas, recent surgical series increasingly favor early intervention to optimize long-term outcomes [2]. The favorable evolution observed in our patient without surgical intervention may be explained by good responsiveness to antiepileptic therapy, absence of lesion progression, and stability of the hemosiderin rim on serial MRI, which likely limited ongoing epileptogenic activity [2].

The natural history of CCMs indicates a high risk of seizure recurrence, estimated at up to 94% within five years following an initial seizure if left untreated [11]. Nevertheless, with appropriate management, long-term seizure control is achievable in a significant proportion of patients. Previous studies report that approximately 70% of patients may become seizure-free following effective treatment strategies [10]. In this context, the five-year seizure-free outcome observed in our patient, along with complete resolution of visual symptoms, highlights that optimized medical therapy alone can be an effective management strategy in carefully selected cases of cavernoma-related epilepsy.

## Conclusions

Cerebral cavernous malformations can manifest with headaches, seizures, or focal neurological deficits. Occipital involvement, although less common, may further complicate diagnosis due to visual symptoms that can mimic migraine with aura. This case highlights the importance of considering structural causes in patients with atypical or treatment-resistant visual symptoms. Early evaluation with MRI and an EEG is essential for accurate diagnosis. This case also shows that carefully selected patients with cavernoma-related visual epilepsy may achieve excellent long-term control with medical therapy alone, underscoring the importance of individualized management.
